# The WHO’s critical bacteria list: scientific response eight years after its implementation and development of an AI-based tool for its monitoring

**DOI:** 10.3389/fphar.2025.1633382

**Published:** 2025-09-11

**Authors:** Juan Eduardo Robledo Almonacid, Christian Lombardo, Mariana Romano, Agustina Quiroga, Paula Cambuli Bianchi, Mauricio Hualpa, Constanza Giai, Xiomara María A. Oviedo, Ramiro Alejo Salgado Mansur, Mariana Guadalupe Vallejo, Cristián Andrés Quintero

**Affiliations:** ^1^ Farmacognosia, Departamento de Ciencias Farmacéuticas, Facultad de Ciencia Químicas, Universidad Nacional de Córdoba, Córdoba, Argentina; ^2^ Instituto de Investigaciones en Ciencias de la Salud-Consejo Nacional de Investigaciones Científicas y Técnicas, Córdoba, Argentina; ^3^ Facultad de Ciencias Exactas y Naturales, Universidad de Buenos Aires, Buenos Aires, Argentina; ^4^ Laboratorio de Biología Celular y Molecular-BioCyM. Universidad Juan Agustín Maza, Mendoza, Argentina; ^5^ Instituto de Investigaciones Biomédicas-INBIOMED-UM Facultad de Ciencias Médicas, Universidad de Mendoza, Mendoza, Argentina; ^6^ Obra Social de Empleados Públicos-OSEP-Mendoza, Mendoza, Argentina; ^7^ Departamento Académico de Ciencias Exactas, Físicas y Naturales, Universidad Nacional de La Rioja, La Rioja, Argentina; ^8^ Unidad de Investigación y Desarrollo en Tecnología Farmacéutica-Consejo Nacional de Investigaciones Científicas y Técnicas, Córdoba, Argentina

**Keywords:** antibiotic, antibiotic resistance, automatic classifier, treatment, WHO

## Abstract

**Background:**

In 2017, the World Health Organization (WHO) issued a global alert identifying 12 bacteria in urgent need of new treatments.

**Main body:**

This study assesses the scientific community’s response to this alert by analyzing original research publications using LLMzCor, an AI-based tool developed and validated by our group. To compare trends, we focused on publications from 5 years before and after the alert, specifically on three bacteria listed in the WHO alert, sorted by priority level: *Acinetobacter baumannii* (Critical), *Shigella* spp (High), and *Neisseria gonorrhoeae* (Medium) and three non-listed as controls (*Rickettsia* spp., *C. trachomatis*, and *C. difficile)*. Articles were classified into three categories: (i) identification of Resistant strains, (ii) development of New treatments, and (iii) Immunization strategies.

**Results:**

Although overall publications increased after the WHO alert, no statistically significant changes were found in the reports of Resistant strains over time. The development of New treatments for the listed bacteria showed a slight increase, between 2% and 10%. Furthermore, Immunization strategies remained relatively unchanged, with less than 2%. Meanwhile, LLMzCor demonstrated robust performance across categories, F1-scores ranging from 0.65 to 0.72 in key classifications, while recall peaked at 0.75, indicating a high capacity to identify relevant articles. These results support the model’s reliability for large-scale automated classification of scientific abstracts.

**Conclusion:**

These findings, supported by LLMzCor, underscore the urgency of a stronger WHO alert and action plans to develop new strategies against bacterial resistance.

## 1 Introduction

Antimicrobial resistance (AMR) is an important global health problem that demands urgent solutions. Infectious diseases remain one of the leading causes of death worldwide, with substantial implications for public health and the global economy. Recent studies estimated that nearly 5 million deaths in 2021 were associated with bacterial AMR, and over 1 million were directly attributable to it. Projections suggest that these numbers could double by 2050 (Naghavi et al., 2024).

Antibiotics are the main therapeutic intervention for the treatment of infections of bacterial origin. The first antibiotic was discovered in 1910, and the field experienced a “golden age” of antibiotic discovery between the 1940s and 1970s. However, in recent decades, the widespread misuse and overuse of antibiotics in human medicine, agriculture, livestock, and pet care have fueled the emergence and spread of resistant bacterial strains. Since the 1940s, humans have been contributing to the increase in resistant strains in all environments, due to the uncontrolled release of antibiotics from clinics into nature, in addition to their widespread use in agriculture, livestock, and house pets (Davies and Davies, 2010).

Bacteria can develop resistance through several mechanisms, primarily via spontaneous mutations during DNA replication and followed by the selection of drug-insensitive mutants that spread vertically, or via horizontal gene transfer between bacteria. This phenomenon, known as antibiotic resistance (AR), allows bacteria to survive and thrive despite antimicrobial treatment. The declining efficacy of antibiotics has triggered multiple international warnings (Diallo et al., 2020; Laxminarayan et al., 2013; McEwen and Collignon, 2018).

In order to discover new and effective treatments for the multiresistant bacteria, researchers are addressing their efforts in several directions (Cook and Wright, 2022; Durand et al., 2019; Wohlleben et al., 2016), not only the traditional antibiotics, but also new approaches (León-Buitimea et al., 2020). The more important categories are ***I- Antibodies*,** where the monoclonal antibodies lead the way*,* and the FDA has already approved more than 100 antibodies for medical treatments (Mullard, 2021). ***II- Antimicrobial Peptides (AMP)***, small peptides (i.e., less than 100 amino acids), some produced naturally from the three domains of life, and also engineered derivatives of AMPs that inhibit microbial growth by disrupting the bacterial cell membrane (Ji et al., 2024). ***III- Antivirulence strategy.*** The key to this strategy is to reduce the virulence of pathogenic bacteria by inhibiting their virulence factors (such as toxins, adhesion proteins, quorum-sensing regulators, and extracellular enzymes) rather than killing the pathogen. This allows the immune system to clear the infection in a more effective way (Iskandar et al., 2022). ***IV- Bacteriophages.*** These microorganisms are viruses that specifically infect bacteria. They are known as molecular biology tools, and they were used as antibacterial agents soon after their discovery, but were quickly superseded by antibiotics. However, they have re-emerged as a potential treatment strategy in the last few years, and have been successfully used against *Salmonella* spp. and *Shigella* spp. among others (Połaska et al., 2019). ***V- Combination strategies***. The use of adjuvants or the combination of different antibiotics is also a useful tool. Adjuvants are designed to inhibit the intrinsic resistance mechanisms by which bacteria evade antibiotics. In addition, combinations of different antibiotics can have a synergistic effect, i.e., they are more effective in combination than each antibiotic used individually (Ejim et al., 2011). ***VI- Natural Products (NP).*** The beginnings of the antibiotic era and most of the antibiotics currently in use are derived from natural products, mainly from bacteria, fungi, and plants. Specifically, plants have been used in various cultures as part of traditional medicine to treat diseases. In recent decades, they have come back into focus, using either plant extracts, essential oils, purified bioactive molecules, or even *in vitro* production of secondary metabolites (Quintero et al., 2022; Vallejo et al., 2023).

To guide and encourage the research and development of new antibiotics, the World Health Organization (WHO) issued an alert in 2017, identifying 12 bacteria grouped into three priority categories: Critical, High, and Medium (World Health Organization, 2017). The list was composed as follows: Critical priority for *Acinetobacter baumannii* and *P. aeruginosa,* High priority for *S. aureus, E. faecium, H. pylori, Campylobacter* spp*, Salmonellae,* and *Neisseria gonorrhoeae*, and Medium priority for *S. pneumoniae, H. influenzae,* and *Shigella* spp.

In 2024, a second alert was released, regrettably listing almost the same set of bacterial species (World Health Organization, 2024). This lack of progress raises concerns about whether the first alert had a meaningful impact on the scientific community. Despite considerable efforts to discover new antimicrobials, the adaptability of bacterial genomes and their rapid mutation rates allow these pathogens to “outpace” our research efforts, making it difficult to find a safe and effective solution.

A comprehensive review of the scientific literature is essential to evaluate progress in AMR research and antimicrobial development. However, manual screening of large databases is time-consuming, subject to bias, and requires extensive training and logistical planning (Cumpston et al., 2019). To address these limitations, Large Language Models (LLMs), like LLaMA, Claude, or ChatGPT, have emerged as powerful tools that can be used for automating literature screening, classification, and information extraction in academic and clinical domains (Galli et al., 2025).

Regarding relevant medical information, the automatic selection, especially for real-time decision-making in clinical practice and also in critical contexts like epidemic outbreaks, can be very beneficial in terms of time saving. Medical doctors currently spend almost 50% of their time in recording and documenting clinical procedures (Galli et al., 2025; Mustafa et al., 2025). In this sense, AI-based tools, like srBERT (Aum and Choe, 2021; MediSearch (2025); Glass Health (2025)), among other LLMs to improve medical documentation review have been developed for that purpose.

GPT-4 is one of the most popular LLMs, which can make classifications based on the article summaries, through prompts or well-structured queries specifying inclusion and exclusion criteria. Several reports confirm the successful use of OpenAI in Systematic Reviews (SR) and meta-analyses, with matching results compared to experienced human coders over 80% (Mustafa et al., 2025) and revealing it as one of the most consistent performers (Delgado-Chaves et al., 2025). Nevertheless, an optimized repeated validation of the LLM, sometimes missing or deficient in certain models (Lieberum et al., 2025), is strongly recommended, focusing on prompt readjustment to achieve a higher accuracy (Delgado-Chaves et al., 2025).

Bacterial resistance has been studied from different approaches through the use of AI tools such as Machine Learning (ML), Deep Learning (DL), non-linear DL, Transference Learning (TL), to identify and classify Antimicrobial Resistance (AR) genes, as suggested by different authors. Nayak et al. (2024) designed and validated aiGeneR 1.0, a non-linear DL model for genomic data processing with high accuracy performance, detecting the *Escherichia coli* resistant genes causing the decrease in antibiotic efficiency through horizontal gene transfer. Recently, the development of ARGai 2.0, a TL based-model with remarkable efficiency, allowed the detection of AR genes and resistant strains in the same microorganism, through the analysis of high-throughput (NGS) GE data (Nayak et al., 2025). On the other hand, Singh and Sodhi (Singh and Sodhi, 2024) used a ML model to improve the selection of the most appropriate treatment, based on the interaction of antibiotic-resistant compounds with target proteins.

**FIGURE 1:**
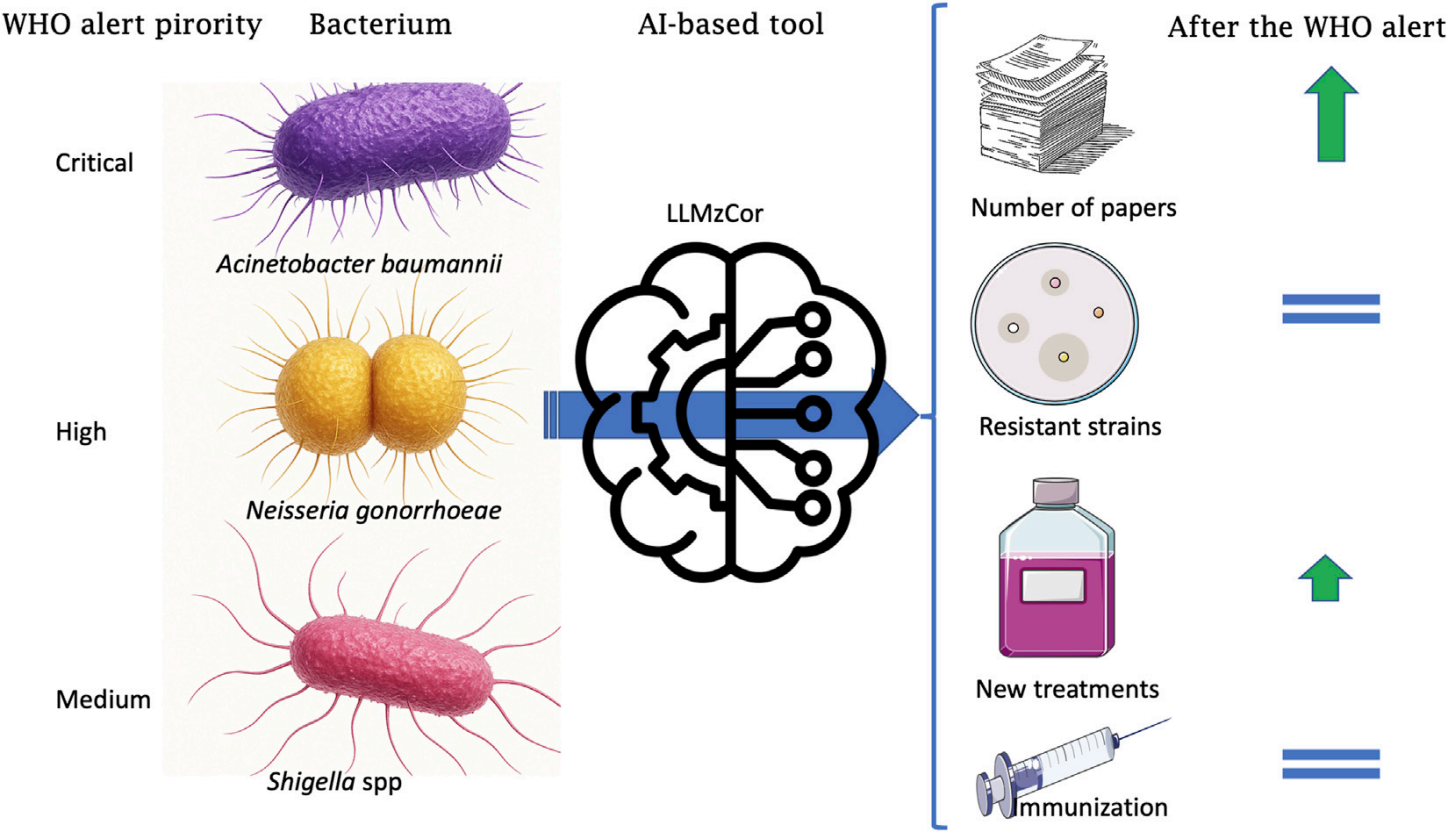
Schematic representation of the results. *Acinetobacter baumannii*, *Neisseria gonorrhoeae, and Shigella* spp., listed as Critical, High and Medium priority in the WHO alert, analyzed with LLMzCor, and the trends in number of original papers, publications on multiresistant bacteria strains, new treatments and new strategies of immunizations against the selected bacteria.

Despite the alert and efforts by the scientific community, advances in the development of new treatments or immunization strategies have not been sufficient to combat AR effectively. Considering the critical situation of AR, the urgent need for rapid development of therapeutic strategies, the importance of updated SR for decision-making in clinical areas, and the growing use of LLMs in writing SRs, the aim of this study is twofold. First, to compare the state-of-the-art of AR and treatment, before and after the 2017 WHO alert on selected priority bacterial pathogens, considered them as indicators, as well as others of current clinical importance. Second, to develop and test an LLM tool to optimize the preparation of comprehensive reviews (Figure 1).

## 2 Materials and methods

### 2.1 Screening strategy

We developed a strategy and performed a search of the literature in PubMed from 2012 to 2022, in order to cover the periods corresponding to 5 years before and after the 2017 WHO alert (World Health Organization, 2017).

The application of Boolean operators (AND, OR, NOT) was utilized to refine the search results. Therefore, the following search terms were utilised: The following search terms should be used to identify relevant literature on the subject: (Therapeutic updates OR Therapeutic innovation OR Drug development OR Antibiotic resistance OR Antimicrobial development) AND (<Bacteria name>).

In order to generate and validate the accuracy of the LLM (see Section 2.3), and to include a manageable number of articles for human reviewers, while achieving an acceptable *n*, we selected one bacterium from each category of the WHO alert list, and its mortality, treatability and resistance to certain types of antibiotics were considered (World Health Organization, 2017). The remaining three non-listed bacteria, which are of regional and global importance, were also included, according to their transmission routes (vectors, sexual transmission, and fecal-oral route). The < Bacteria name > term corresponds to one of the following pathogens, namely, Priority 1: CRITICAL *A. baumannii*; Priority 2: HIGH *N. gonorrhoeae*; Priority 3: MEDIUM *Shigella* spp.; and the three bacteria not listed in the alert: *Rickettsia* spp., *Chlamydia trachomatis,* and *Clostridium difficile*.

### 2.2 Selection criteria

We included studies that met the following criteria, keeping in mind the aim of this article. These articles were considered for inclusion in the review: 1) original research articles published within 5 years before or after the alert, that included the names of the bacteria mentioned above; 2) *in vitro* and *in vivo* preclinical assays; 3) *ex-vivo* studies using clinical samples; 4) studies on hospital environmental samples and biomedical devices; 5) epidemiological studies where a resistant strain was characterized and reported; 6) phylogenetic and clonal studies involving resistant bacteria leading to characterization of antimicrobial-resistant (AR) strains and/or the identification of new treatments.

On the other hand, the exclusion criteria included reviews, meta-analyses, editorial, comments, opinions, and letters to the editor that did not report antimicrobial resistance (AR).

### 2.3 Database construction

To carry out this project, freely accessible data from PubMed were used. The first classification was according to their publication date, dividing them into pre-alert 2 (25/02/2012–25/02/2017) and post-alert (26/02/2017–25/02/2022). A sample of the database *n* = 1800, was used to test set analysis (see section 2.5). Titles and Abstracts were screened independently by two reviewers against predefined eligibility criteria. Discrepancies between reviewers were resolved through discussion or consultation with a third reviewer. In order to validate the accuracy of the model as a classifier, the abstracts were pre-validated to ensure they met the minimum requirements for analysis by the language model, including the presence of an abstract. Records containing special characters within the abstract were preprocessed to avoid misinterpretation by the LLM. This preprocessing involved converting the content into standardized plain text, thereby enabling more effective input into the model.

Subsequent automated analysis by the LLM, the output was post-processed to transform the information into one of the following classification categories: 1) Reporting of resistant strains, 2) New treatments, 3) Immunization, and 4) None, where Category 4, contains any report that does not fall into other categories. For Category 2, New treatments, Sub-categories mentioned in the Introduction were also included: a) Natural products, b) Peptides, c) Designer drugs, d) Antibodies, e) Bacteriophages, f) Antivirulence strategies, and g) New combination of antibiotics, as well as h) Off-label (use), or i) Physicochemical treatment. These two subcategories were defined as: h) use of a pharmaceutical agent for an unapproved indication or in an unapproved age group or at a different dose, duration, or route of administration (Gazarian et al., 2006) and i) use of physicochemical agents in antimicrobial therapy.

### 2.4 Application of Large Language Models

For the classification task, we employed the Mixtral 8 × 7B model (Jiang et al., 2024), a pre-trained Sparse Mixture of Experts (MoE) architecture developed by Mistral AI. This model was selected due to its superior inference speed, open-source availability, and high accuracy scores reported in benchmark evaluations. These characteristics made Mixtral particularly suitable for processing large volumes of text efficiently while maintaining reliable classification performance.

### 2.5 Performance evaluation

To assess the model’s effectiveness, we utilized a test dataset (*n* = 1800), manually labeled by members of the research group (reviewers), serving as the ground truth for classification categories. While Recall was selected as the primary evaluation metric—given that missing relevant articles (false negatives) was considered more critical than including irrelevant ones (false positives)—Precision was also reported to reflect the model’s ability to correctly identify relevant categories without increasing the generation of false positives.

Additionally, F1-score was calculated to provide a balanced view of both Precision and Recall. The classification outputs of the Mixtral model were directly compared to the human expert annotations to measure concordance and identify discrepancies through an Accuracy Score, calculated as the number of correct predictions over the total number of evaluated samples.

To further support this evaluation, Class-specific Confusion Matrices were generated, allowing for detailed analysis of error patterns and identification of categories with a higher likelihood of misclassification. This combination of metrics and visualizations facilitated an integrated assessment of the classification system’s performance.

Furthermore, for Category 4 (None), two authors independently reviewed 20% of the abstracts to assess the reliability of the LLMzCor model in detecting non-relevant reports.

### 2.6 Quantification of the impact of the 2017 WHO alert

Data analysis was developed by year, considering the two periods, pre- and post-alert. First, total publications per bacterium were determined by year and by period. Additionally, their proportions were calculated to determine the predominance of each species reported in the publications by year and period. Then, to eliminate the influence of the increase in general publications about the bacteria, the number of articles was normalized by dividing it by the total number of articles per year or period (pre- and post-alert), per bacterium. This procedure was applied to determine the proportions of publications for each category [1) Reporting of resistant strains; 2) New treatments; 3) Immunization], individualized by bacteria, related to total publications by year, and regarding total publications of each period (see Formulas 1–8 in Supplementary Material). When comparing two percentages belonging to pre- and post-alert periods, a proportion difference with a confidence interval of 95% was estimated through a *Z*-test and considering p < 0.05 as statistically significant. Proportions of the subcategories for New treatments were also calculated. Epidat 4.2 was used for these comparisons.

## 3 Results

### 3.1 Search and screening

**FIGURE 2:**
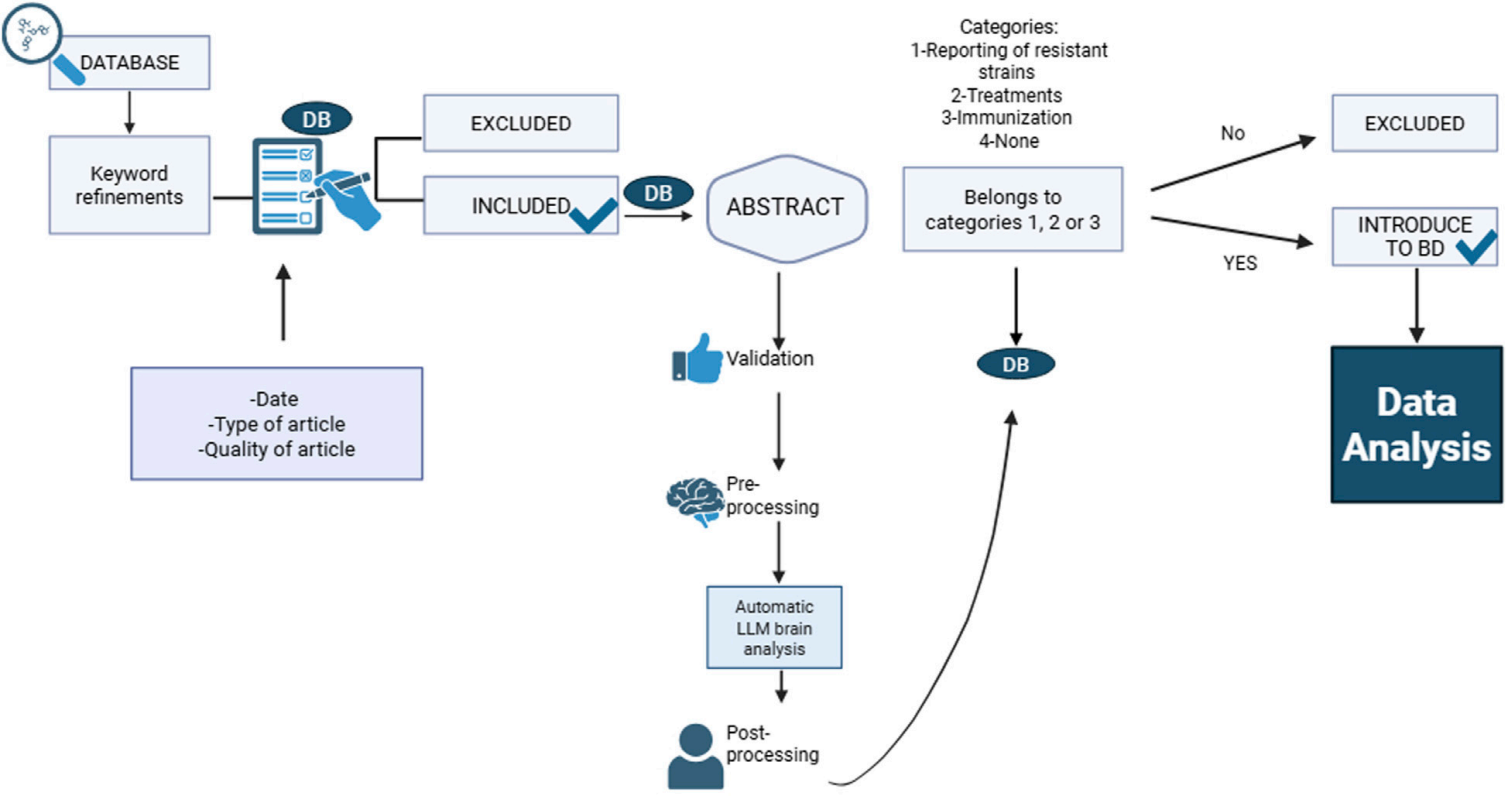
Flowchart of automatic classification of LLMzCor.

The search was conducted between May 2024 and May 2025. A total of 40,308 articles were identified with no duplicates. After depuration according to inclusion/exclusion criteria, 34,252 titles and abstracts remained. Finally, after a second round of revision, 29,231 articles were included in the LLMzCor. This flowchart is shown in Figure 2.

### 3.2 LLMzCor performance

Validation of LLMzCor demonstrated a good performance. Adjusting the inclusion criteria recognized as “true” after a second round of review, the score was 0.83 (number of corrected classifications/total of evaluated publications).

Besides, based on the model’s predictions and the corresponding human classifications, a confusion matrix was constructed to analyze the agreement and discrepancies between both sources of annotation. Two distinct normalization strategies were applied to the confusion matrix in order to facilitate the interpretation of classification performance at the class level.

**FIGURE 3:**
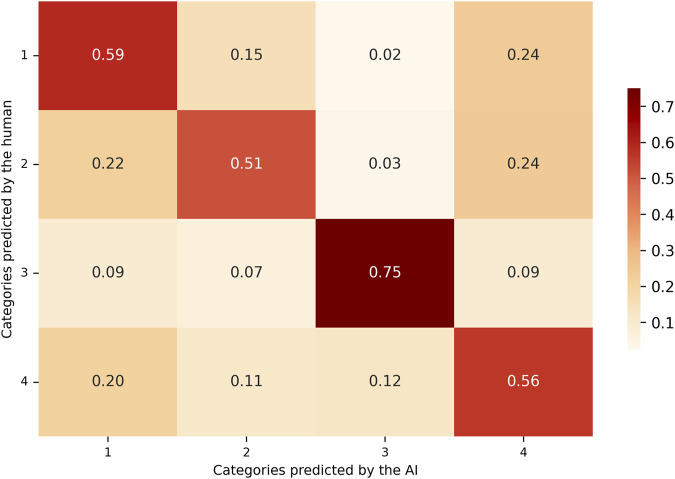
Recall-based confusion matrix comparing AI-predicted and human-assigned categories. Values are row-normalized and represent the proportion of true positives over all actual instances per class. This metric reflects the model’s sensitivity, indicating how well the LLM detects relevant articles within each category.

As illustrated in Figure 3, the matrix values were normalized by row, dividing each cell by the total number of instances in the corresponding true category (as defined by human annotation). Each cell thus represents the proportion of true examples of a given class that were classified into each predicted category by the model. The diagonal elements correspond to the recall for each class, indicating the model’s ability to correctly identify true instances of that category.

**FIGURE 4:**
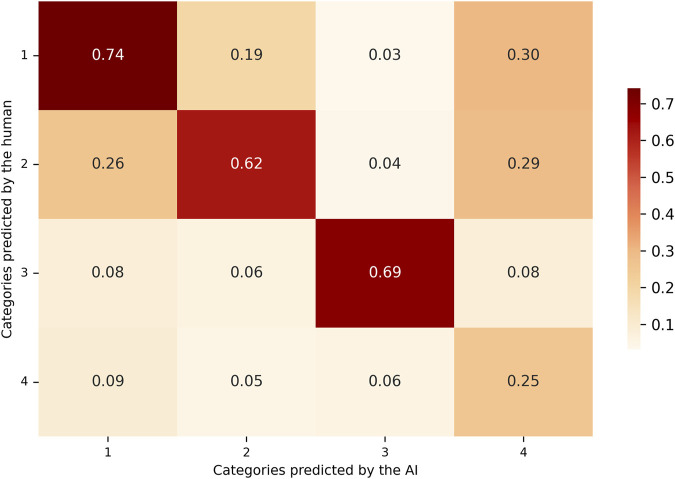
Precision-based confusion matrix comparing AI-predicted and human-assigned categories. Values are column-normalized and represent the proportion of true positives over all predictions made by the LLM for each class. This metric indicates the reliability of the model’s positive classifications.

Column-normalized matrix (Precision-oriented): the matrix values are normalized by column, dividing each cell by the total number of instances predicted by the model for that specific category, as shown in Figure 4. Each cell in the matrix represents the proportion of model-predicted examples that actually belonged to the true category assigned by the human reviewers. The diagonal values in this matrix reflect the precision of each class, indicating how reliable the model’s positive predictions were for each category.

**FIGURE 5:**
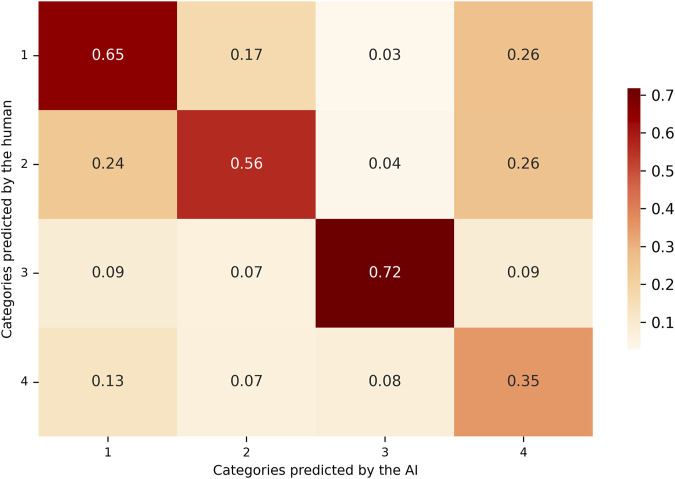
F1-score confusion matrix combining precision and recall. Values reflect the harmonic mean of precision and recall for each category, offering a balanced metric to evaluate overall classification performance. Higher scores suggest more consistent agreement between the LLM and human reviewers.

In Table 1, recall scores showed similar values for categories 1, 2, and 4. In these categories, the model successfully identified approximately 50% of the true cases, as established by the human annotations. In contrast, category 3 exhibited higher recall, indicating that the model correctly identified a greater proportion of the instances assigned to this category by the human reviewer. Precision scores showed that for the first three categories, when the model predicted a specific category, the classification was largely consistent with the human annotation. A reduction in precision was noted in category 4, primarily due to an increase in false positives (FP). In these cases, the model frequently assigned category 4, while the human annotator categorized the same instances under one of the remaining three categories. Category 3 demonstrated the highest F1 score (Figure 5) among all categories, reflecting a more balanced performance in terms of both precision and recall.

**TABLE 1:** Summary of the results of the evaluation metrics, Recall, Precision, and F1 score per category.

Category	Recall (%)	Precision (%)	F1 score (%)
1) Reporting of resistant strains	59	74	65
2) New treatments	51	62	56
3) Immunization	75	69	72
4) None	56	25	35

A quality control was performed on a sample of this category (20% of the articles of every species) by two reviewers, who concluded that this classification was run in accordance with the specified prompts.

From the total articles analyzed by LLMzCor, 55.9% were classified into the 4th category (None). This category was also submitted to a quality control on a sample (20%) by reviewers, with remarkable results. Articles whose aim was on epidemiological or molecular biology studies, new diagnostic techniques, comorbidity description, after-effects of infection, hospital pharmacotherapy protocols (except for those where a new treatment was used), among others, fall into this category. Some justifications formulated by LLMzCor are shown as examples, for *Acinetobacter baumannii*, *N. gonorrhoeae,* and *Shigella* spp, respectively:

“The paper discusses the genomic sequence analysis of *Acinetobacter* *baumannii*, revealing a putative Acid Phosphatase (AcpA). The recombinant protein was expressed in *E. coli*, and its properties were studied, but no new treatment, multi-resistant bacterial strain, or immunization process was discovered or discussed.” (Smiley-Moreno et al., 2021).

“The paper discusses the purification, enzymatic degradation, and separation of peptidoglycan fragments by HPLC, and preparation of samples for mass spectrometry identification, without mentioning new treatments, multi-resistant bacterial strains, or immunization.” (Schaub and Dillard, 2019).

“The paper discusses a rapid, convenient, point-of-care, and accurate identification method for virulent *Shigella* sonnei, but it does not involve new treatments, immunization, or multiresistant bacteria strains.” (Wang et al., 2022).

### 3.3 Quantification of the effect of the WHO alert on the scientific research

In order to evaluate the effect of the alert on the scientific community, scientific publications of six selected bacteria were used as a parameter for the evaluation, three of them belonging to the list, with one from each priority, *A. baumannii, N. gonorrhoeae,* and *Shigella* spp.*,* and three used as an external control, *Rickettsia* spp., *C. trachomatis,* and *C. difficile.*

#### 3.3.1 Increase in the original publication number

In the context of the present study, a total of 10,047 articles reported on *C. difficile*, which had the highest publication trend, followed by 8,106 articles related to *A. baumannii*, while 3,551 articles for *N. gonorrhoeae* position it as the least reported in the 10-year window. A comparative analysis of the extant literature reveals that the total publications pertaining to the remaining bacteria, *Shigella* spp. and *C. trachomatis*, exhibited a high degree of similarity. However, a slight increase in the number of publications concerning *Rickettsia* spp. was observed.

**FIGURE 6:**
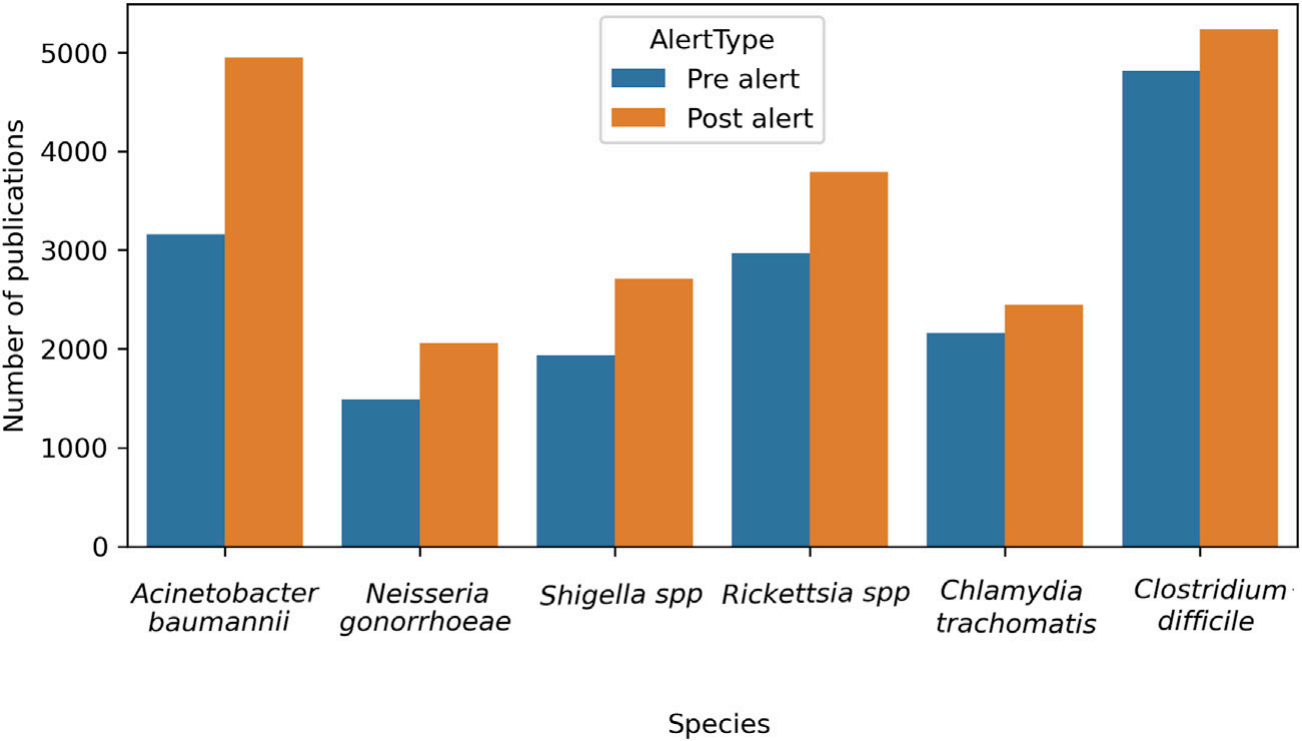
Evolution of the total number of original papers for each bacteria pre- and post-alert. Considering February 2017 as the reference point, when the alert was issued, a time frame of 5 years before (pre-alert) and 5 years after (post-alert) was analyzed.

The first measured item of interest was the total number of original papers published in the time frame, encompassing 5 years before and 5 years after the alert was issued. Figure 6 shows the comparison of the total number of papers for individual bacterium within both periods. An increase was detected in all the bacteria reports analyzed, and when the percentage increase was analyzed, *A. baumannii* tops the list (56.5%), followed by *Shigella* spp. (39.8%) and *N. gonorrhoeae* (38.3%), while *Rickettsia* spp. presented a moderate rise (27.4%), and *C. trachomatis* (13.1%) and *C. difficile* (8.7%) exhibited the lowest (see Formula 1, Supplementary Material).

From a different perspective, when the volume of publications for species was analyzed, *C. difficile* had the highest rate and *A. baumannii* claimed the second place, in both 5-year windows. When comparing pre- and post-alert periods, the *C. difficile* rate decreased [from 29.1% to 24.7%, (0.035, 0.053; Z = 9.61; p < 0.0001] and *A. baumannii* increased from [19.1%–23.3%; (−0.051, −0.034; Z = −9.95; p < 0.0001)]. The remaining species presented similar but small proportions, hence, they did not stand out, even though for most of them statistically significant differences were evident between one period and another (See Formula 2 and Table 1, Supplementary Material).

**FIGURE 7:**
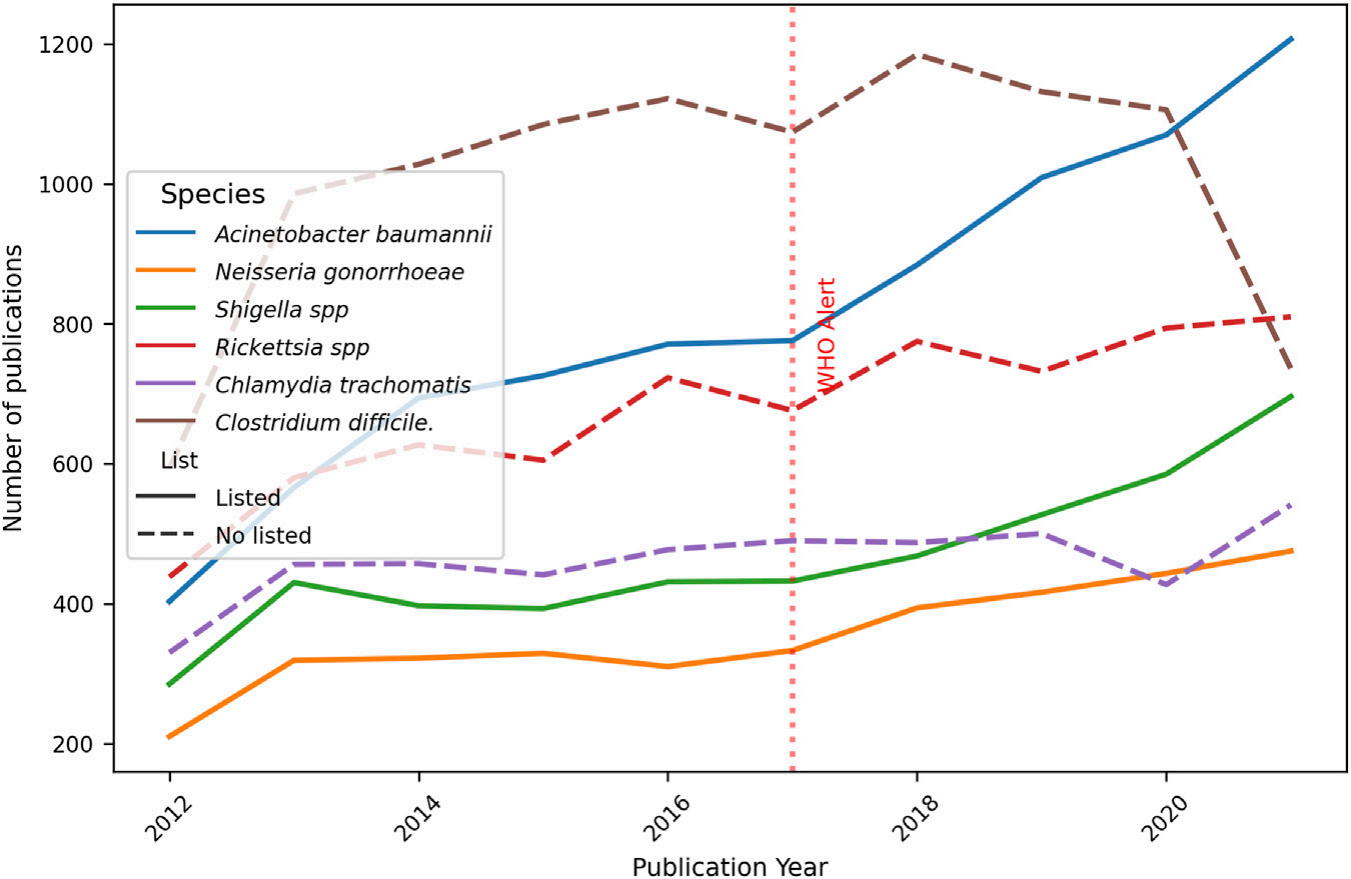
Evolution of the total number of original papers for each bacteria. Ten-year progression of the number of publications about resistance topics on selected bacteria, listed and no-listed in 2017 WHO alert. The red dotted perpendicular line indicates the WHO alarm emission. The continuous line indicates the bacteria was listed in the alert, the dotted line is for bacteria not listed.

Furthermore, the number of publications per year is discriminated against in order to detect trends over time. In Figure 7, a generalised increase in the publications for all six bacteria examined is evident, with a slight diminished tendency starting in 2020 for some species. When analyzing each bacterium individually, a consistent increase in the number of publications was found year-on-year.

#### 3.3.2 Antibiotic resistance (impact on the frequency of new resistant strains description)

**FIGURE 8:**
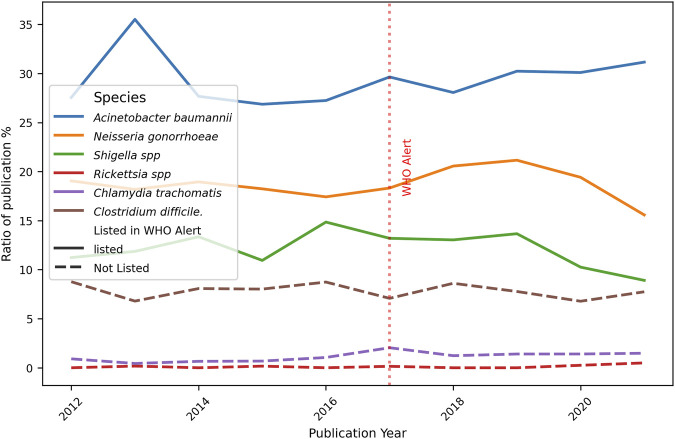
Ratio of number of publications on Resistance bacteria strains and total publications per year. The graph shows the number of publications focusing on the new resistant strains described divided for the total number of papers X 100, for each bacteria and year of publication. The red dotted perpendicular line indicates the WHO alarm emission. The continuous line indicates the bacteria was listed in the alert, the dotted line is for bacteria not listed.

The second point of the current research was to evaluate the report of strains resistant to standard-of-care treatment. In this regard, publications were categorised by year and by period. As demonstrated in Figure 8, the ratio of publications pertaining to resistant bacterial strains to the total number of publications per annum is indicated (see Formula 3, Supplementary Material). A slight rise in the reports of resistant strains was noted over time for all the microorganisms studied, except for *Shigella* spp., which presented a decreased trend, and *C. difficile*, whose reports exhibited low percentages and, at first glance, barely changed over the whole period studied. *A. baumannii* had the highest percentage of publications related to the total by year, slowly decreasing towards the end of the period. In contrast, *Rickettsia* spp. and *C. trachomatis* had the lowest rates. For all the remaining species, reports of resistant strains ranged between 7% and 20% over the 10-year period.

**FIGURE 9:**
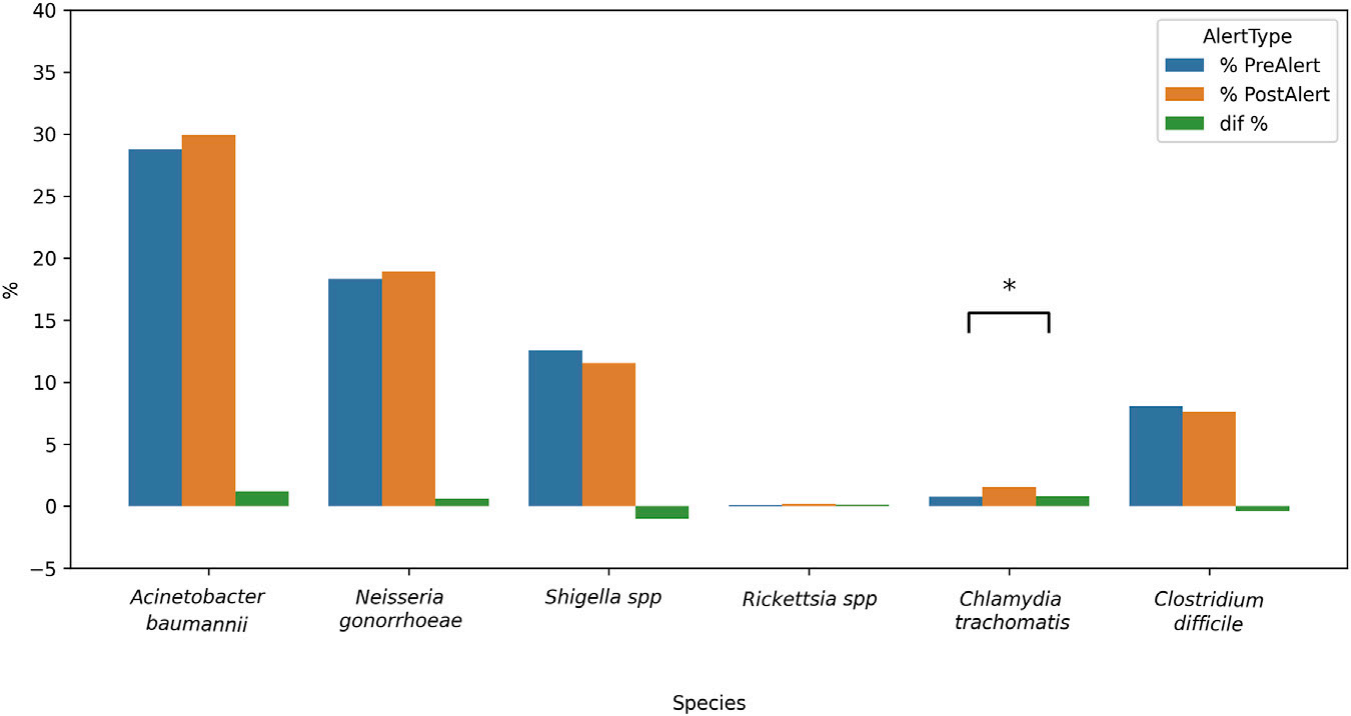
Inter-period percentage of change in publications on Resistant bacteria strains. The difference pre and post alert was calculated for each bacteria. From left to right, listed in the alert as Critical priority, High priority or Medium priority, plus 3 bacteria non listed in the alert. Blue bars = 2012–2016; orange bars = 2017–2021; green bars = percentage difference; *p < 0.05.

The subsequent analysis concentrated on the periods both prior to and following the alert. Figure 9 presents the rate of resistant bacterial strains reported by period (see Formula 4, Supplementary Material). Only *C. trachomatis* percentage differences between pre- and post-alert periods were statistically significant: 0.77% [-0.014, −0.002; Z = −2.454] p < 0.005. All the remaining bacteria rate differences were not statistically significant (pre- and post-alert period rates are shown in parenthesis): *A. baumannii* exhibited the highest percentages (28.8%–30%) which is consistent with the previous result, and similar observation holds for *N. gonorrhoeae* (18.3%–19%), *Shigella* spp (12.6%–11.5%) and *C. difficile* (8%–7.6%). Low values were found for *Rickettsia* spp. and *C. trachomatis,* whose percentage changes were not significant.

#### 3.3.3 Therapeutic strategies (positive impact on the new treatment discovery after the alert)

The primary objective of the alert was to encourage the scientific community to conduct research and identify novel treatments for these bacteria.

**FIGURE 10:**
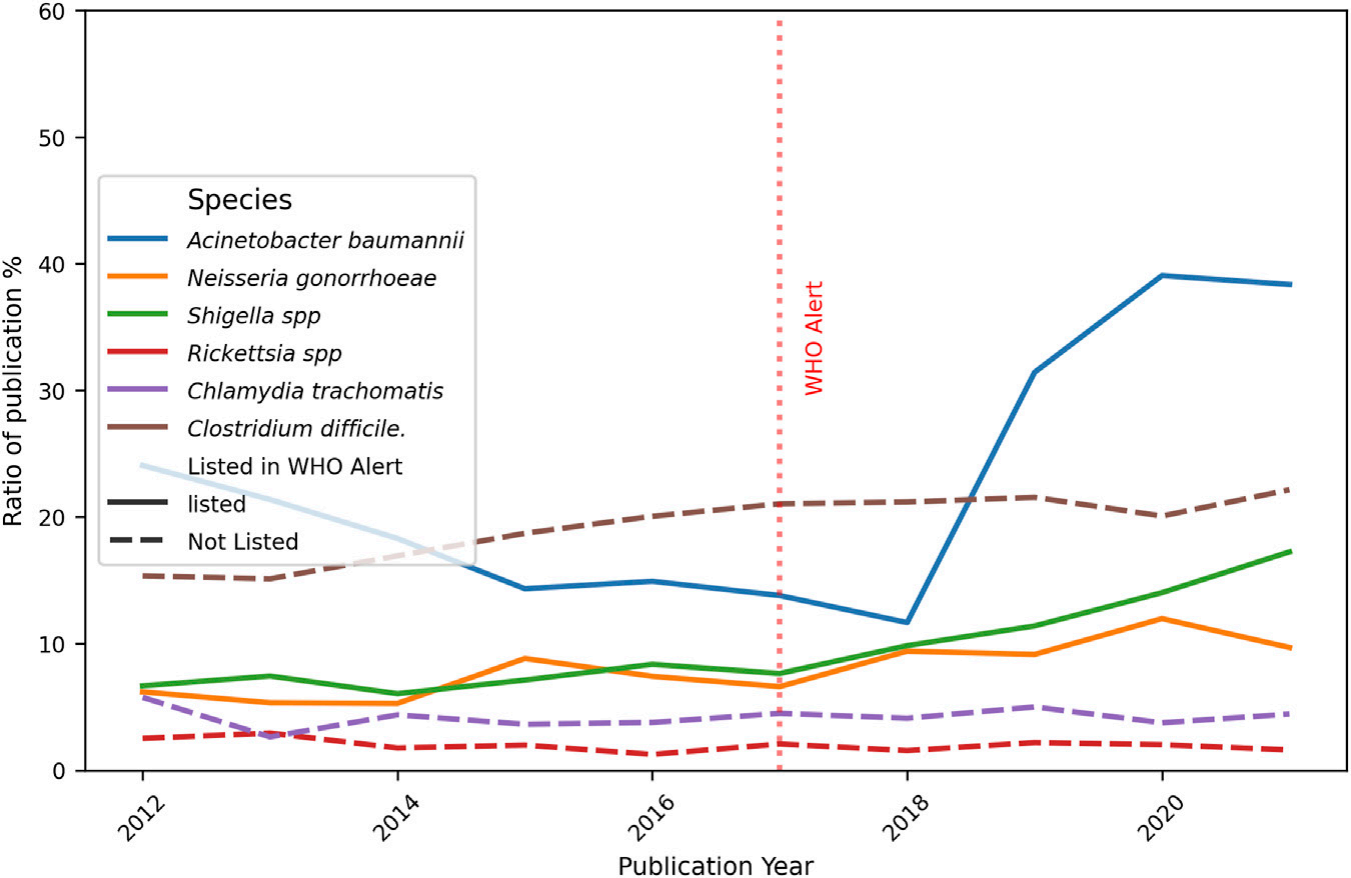
Ratio of number of publications on New treatments and total publications per year. The graph shows the number of publications focusing on the new treatments divided for the total number of papers X 100, for each bacteria and year of publication. The red dotted perpendicular line indicates the WHO alarm emission. The continuous line indicate the bacteria was listed in the alert, the dotted line is for bacteria not listed.

As illustrated in Figure 10, the publication rate of *A. baumannii* slightly decreased, with a turning point at the beginning of the post-alert period, and subsequently largely recovered (see Formula 5, Supplementary Material). Furthermore, an increase in the reports on new treatments for *Shigella* spp. and *N. gonorrhoeae* was observed towards the end of the study period, with this trend being more marked in the former. Moreover, the evolution over time of new treatments for unlisted bacteria did not show significant changes. Hierarchical clustering analysis revealed that the highest levels were found for *C. difficile*, followed by *C. trachomatis*, with *Rickettsia* spp. in last place.

**FIGURE 11:**
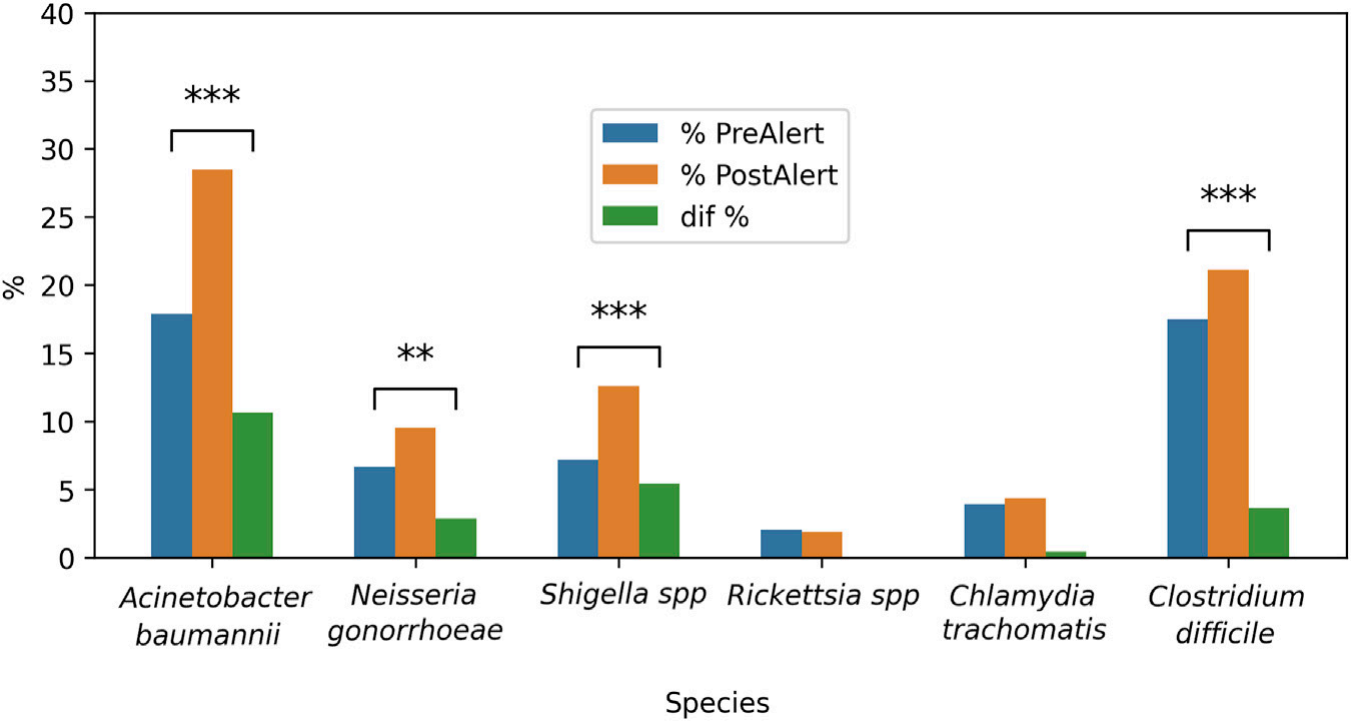
Inter-period percentage of change in publications on New treatments against selected bacteria. The difference pre- and post-alert was calculated for each bacteria. From left to right, listed in the alert as critical priority, high priority or medium priority, plus 3 bacteria non listed in the alert. Blue bars = 2012–2016; orange bars = 2017–2021; green bars = percentage difference; **p < 0.005; ***p < 0.0001.

As demonstrated in Figure 11, a by-period analysis revealed a notable increase in reports concerning new treatments for *A. baumannii*, *Shigella* spp. and *N. gonorrhoeae* in the post-alert period (see Formula 6, Supplementary Material). This finding is corroborated by the statistically significant differences observed.: 10.62% [−0.125, −0.088; Z = −10.87] p < 0.0001; 5.41% [−0.071, −0.37; Z = −5.794] p < 0.0001, and 2.87% [−0.047, −0.011; Z = −3.05] p < 0.005, respectively. Fortunately, the *C. difficile* percentage also raised, 3.64% [−0.052, −0.021; Z = −4,616] p < 0.0001.

**FIGURE 12:**
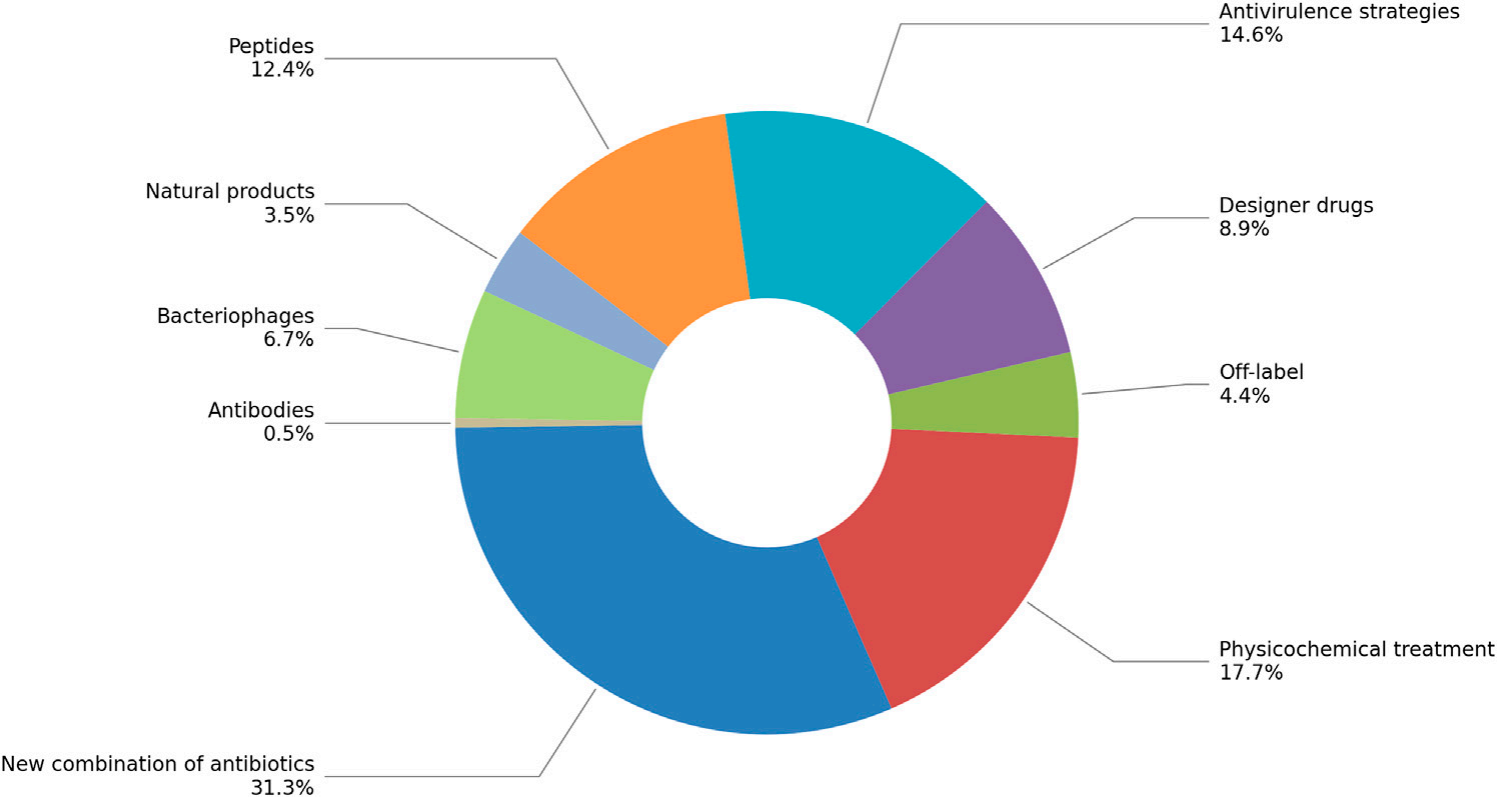
Distribution of subcategories on New treatments publications for all bacteria studied, expressed as percentages.

As a preliminary analysis of the type of treatment, we performed a deeper classification according to the sub-categories mentioned in the Introduction. Figure 12 shows the distribution of new treatment publications. Overall, the main subcategory obtained was g) New combination of antibiotics (31.3%), followed by i) Physicochemical treatments (17.6%); f) Antivirulence strategies (14.6%); b) Peptides (12.4%). Surprisingly, c) Designer drugs (8.9%) and e) Bacteriophages (6.7%) were in 5th and 6th place, respectively, surpassing h) Off-label (4.4%) and a) Natural Products (3.5%), while d) Antibodies (0.5%) had the lowest rate. For subcategory g), reviewing of a sample (20%) for each bacterium suggested the use of clinically known antimicrobial agents in combination in order to apply a synergistic strategy against a resistant strain or reduce the doses for a safer therapy. In subcategory i), gases, UV-light, or oxidant agents, among others, are proposed as potential therapies.

#### 3.3.4 Immunization: the vaccine development at a plateau

Immunization strategies are sought after due to their reduced potential for the development of resistance when compared with bacteria. Consequently, it was imperative to be cognizant of the advances pertaining to immunizations in the bacteria under scrutiny.

**FIGURE 13:**
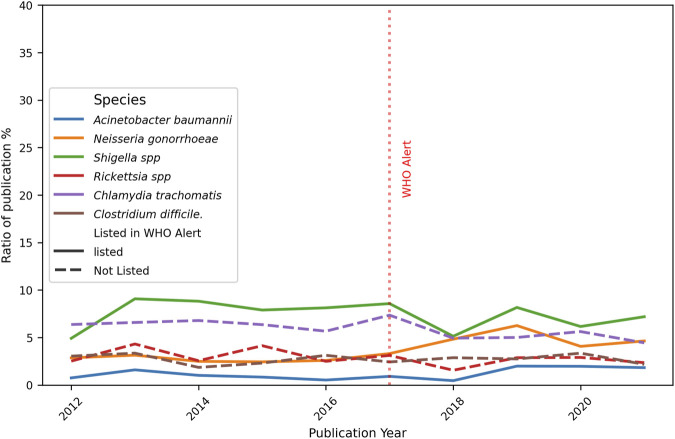
Ratio of number of publications on new strategies of Immunizations and total publications per year. The graph shows the number of publications focusing on the new immunization strategies divided for the total number of papers X 100, for each bacteria and year of publication. The red dotted perpendicular line indicates the WHO alarm emission. The continuous line indicates the bacteria was listed in the alert, the dotted line is for bacteria not listed.

Figure 13 shows the percentage of reports on new immunization therapies across the 10-year period (see Formula 7, Supplementary Material). Throughout the duration of the study, low levels were consistently observed, with these levels remaining below 10% for each bacterial strain. New immunization strategies were observed for *Shigella* spp. and *C. difficile* were the highest, followed by *C. trachomatis*. For *A. baumannii*, meanwhile, reports on these strategies were rarely published, standing at minimal rates, although they increased toward the end of the post-alert period, as did rates for *N. gonorrhoeae*.

**FIGURE 14:**
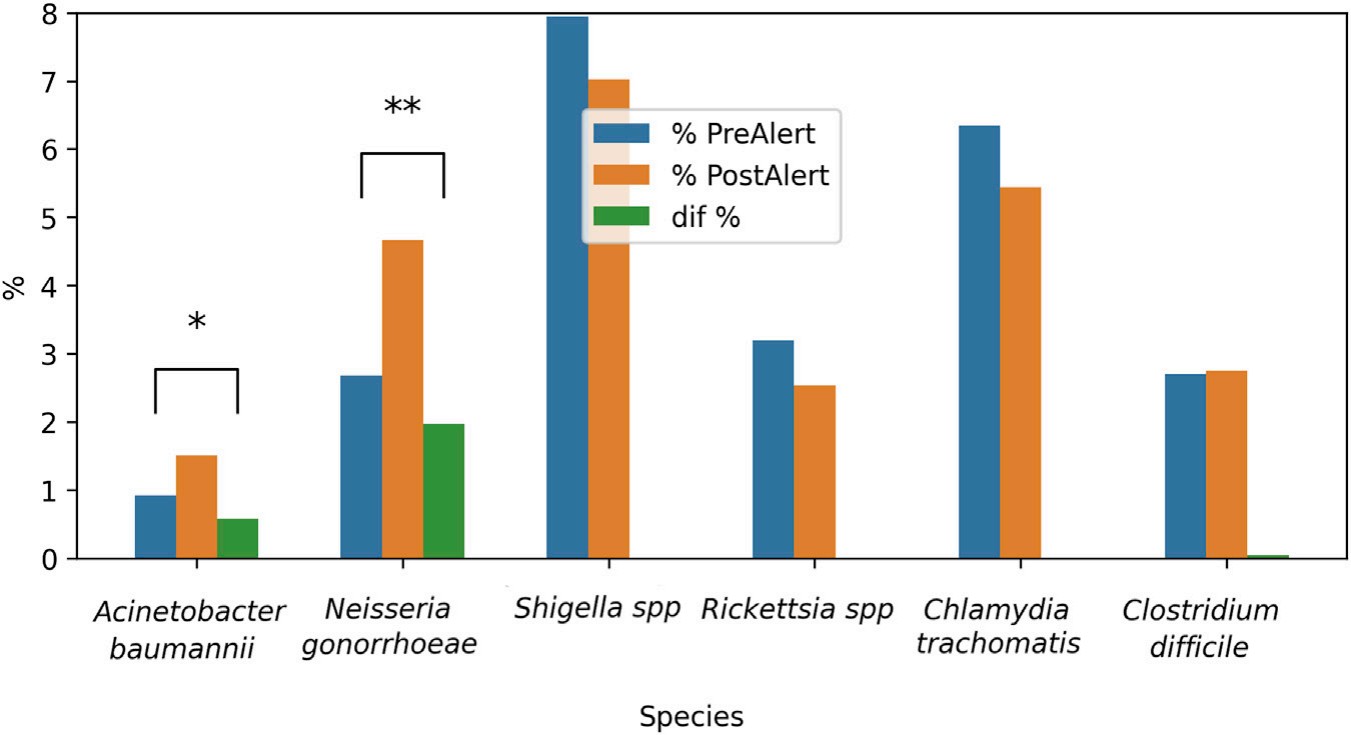
Inter-period percentage of change in publications on new Immunizations strategies against selected bacteria. The difference pre- and post-alert was calculated for each bacteria. From left to right, listed in the alert as critical priority, high priority or medium priority, plus 3 bacteria non listed in the alert. Blue bars = 2012–2016; orange bars = 2017–2021; green bars = percentage difference; *p < 0.05.

Complementary, Figure 14 presents the same reports considering pre- and post-alert periods (see Formula 8, Supplementary Material). Only for *N. gonorrhoeae* and *A. baumannii* significant changes were observed, 1.97% [-0.032, −0.007; Z = −3.024] p < 0.005, and 0.58% [-0.011, −0.001; Z = −2.268] p < 0.05, respectively. The rates of the remaining microorganisms remained unchanged, according to the statistical estimates made (pre- and post-alert rates are shown in parenthesis): *Shigella* spp. (7.95%–7.02%); *Rickettsia* spp. (3.20%–2.53%); *C. trachomatis* (6.34%–5.44%) and *C. difficile* (2.70%–2.75%).

## 4 Discussion

In this work, the implementation of the AI assistant significantly streamlined the classification process, enabling a more efficient review of the literature, with a remarkable score (0.83), taking into account that a perfect match between the automated tool and the manual researcher review is ambitious, since human criteria may still have discrepancies (Delgado-Chaves et al., 2025). This performance was possible by means of a redefinition of the eligibility criteria; therefore, a constant review of the parameters (prompts) required by the LLM is desirable. LLMzCor demonstrated a robust and consistent performance across Precision, Recall and F1 scores. The discrepancies observed between the model’s predictions and the categories assigned by human reviewers do not necessarily indicate poor system performance. In certain cases, the articles exhibit features that hinder unambiguous classification, even for expert human evaluators. In such situations, the model may capture subtle patterns or contextual cues that allow for a more nuanced categorization—one that, although differing from human annotation, may be equally valid or even more appropriate. To improve the quality of classifications generated by LLMzCor, several strategies could be explored. One such approach involves the use of multiple LLMs rather than a single model, implementing a voting-based decision system. This ensemble strategy introduces multiple “voters” into the classification process, increasing the diversity of perspectives on each case. As a result, individual variability may be reduced, enhancing the robustness and Precision of classification decisions.

Beyond technical optimization, this study aligns with a broader transition in the way research is conducted. The current challenge is no longer the lack of information, but rather the ability to navigate, process, and synthesize large volumes of scientific data efficiently. LLMs enable direct interaction with textual content, facilitating the detection of informational patterns, identification of evidence gaps, and generation of novel hypotheses in reduced timeframes. The rapid improvement of LLM performance suggests that repeating this same study in the near future would likely yield even better results. This ongoing advancement enables the development of dynamic, adaptive systems capable not only of reading on behalf of researchers, but also of supporting strategic reasoning and evidence-based scientific decision-making.

Building on these insights, and in alignment with current global health priorities, the following analysis focuses on antibiotic resistance alert issued by the WHO in 2017 as a framework to explore publication trends and research efforts over the last decade. This alert on 12 bacteria that urgently require antibiotic development due to their increased resistance is aimed at researchers and pharmaceutical companies to incentivize the R&D of new antibacterial agents, providing clear directions on which bacteria must be prioritized.

In the present study, a selection of three bacteria from each of the WHO alert’s priority categories was made, in addition to three non-listed pathogens that are of clinical importance. A comprehensive analysis of the total publications from 2012 to 2022 reveals that *C. difficile* had the leading role (10,047 articles). It is a spore-forming, obligate anaerobic Gram-positive bacilli bacterium, having nosocomial prevalence. It can colonize the gastrointestinal tract and the disruption of gut microbiota by antibiotic therapy facilitates its invasion, which is the most common clinical manifestation of diarrhea, affecting hospitalized patients. Probably due to this deep concern, numerous reports were identified and gained predominance on total publications per period (29.1% and 24.7%, Supplementary Material, Table 2). On the other hand, *A. baumannii* is an extracellular, strictly aerobic, nonmotile, Gram-negative coccobacillus. Even when it has been implicated in a wide spectrum of infections, the most prevalent is nosocomial. The principal site of colonization is the respiratory tract, becoming dangerous in patients in intensive care with mechanical ventilation. Indeed, the second place of total reports was for *A. baumannii,* belonging to Critical priority (8,106 articles). This is also stated by its importance on total publications per period (19.1% and 23.3%, Supplementary Material, Table 2). At the other extreme, 3,551 articles were found for *N. gonorrhoeae* (High priority category), a cocci-like morphology, Gram-negative, strictly aerobic, and facultatively intracellular microorganism. It can colonize the urethra, endocervix, rectum, pharynx, and anus. Being the causal agent of gonorrhea, the second most prevalent sexually transmitted infection in the world, it can be worrying that a lower number of articles were detected. When publications for each bacterium were analyzed by period (Figure 6), all of them increased, mainly *A. baumannii* (+56.3%), *N. gonorrhoeae* (+38.3%), and *Shigella* spp. (+39.8%). These encouraging facts respond to the WHO alert, and mean that, at least, general publications on the three listed bacteria increased from pre-to post-alert period, even when they present a low number of publications in total analyzed by LLMzCor. That applies to *N. gonorrhoeae* and *Shigella* spp. The latter are nonmotile intracellular Gram-negative rods. They are the causal agent of shigellosis, an invasive condition that affects the colon and rectum, producing epithelial invasion with significant fluid and electrolyte losses. Regarding the three non-listed bacteria, they were the ones whose reports increased the least. This can be interpreted as a focusing of investigations on the pathogens alerted by the WHO. From them, *Rickettsia* spp. exhibited a moderate rise (+27.4%). They are Gram-negative obligate intracellular bacteria in vertebrates, usually mammals, and are also associated with bloodsucking arthropods such as fleas, lice, or ticks. The most important diseases they cause in humans are typhus fever, spotted fever rickettsiosis (also called Rocky Mountain spotted fever), and ehrlichiosis. These local etiological characteristics perhaps make them less attractive as an aim of study. Secondly, *C. trachomatis* raised only 13.1%. It is also a Gram-negative, obligate intracellular bacterium that is restricted to humans. It is the major causative agent of bacterial sexually transmitted diseases and preventable blindness worldwide. Non-treated infections can result in urethritis, cervicitis, epididymitis to trachoma, lymphogranuloma venereum, pelvic inflammatory disease, tubal obstruction, ectopic pregnancy, and infertility. *Clostridium difficile,* meanwhile, showed only an 8% increase even though its reports were numerous. Regarding the analysis by year, the total number of original papers for all bacteria showed a constant growth over the years, with a higher increment in the case of *A. baumannii* (Figure 7). The remaining five bacteria showed a deceleration of the growth or a slight decrease in the last year of the post-alert period. The starting point of the decrease for some bacteria was in 2020, consistent with the advent of the COVID-19 pandemic, when numerous scientific groups changed their main focus of research towards SARS-CoV-2, which is our main hypothesis to explain this tendency.

Considering the percentage of papers on the description of New Resistant Strains, our results showed a very small increase over the 10-year period (Figure 8), for *A. baumannii,* while bacteria such as *Neisseria gonorrhoeae* (High Priority) and *Shigella* spp. (Medium Priority) exhibited an almost constant percentage (between 10% and 20%), indicating the regular finding of new strains showing resistance to the regular treatments. *Clostridium difficile* reports exhibited low percentages with no changes over time, and this fact contrasts with the numerous total publications counted for this bacterium. A possible explanation is that most reports focused mainly on epidemiological data and topics other than resistant strains. It is noteworthy that differences in pre- and post-alert (Figure 9) strengthen these findings, since *Acinetobacter baumannii* had a marked increase in the total number of original papers in the post-alert period, which diminished the normalized differences. This is highly relevant since it exhibited multiple mechanisms of resistance, including carbapenemases and beta-lactamases production, overexpression of efflux pumps, loss or alteration of porins, and mutations in gyrase/topoisomerase. These accumulated mechanisms make many strains of *A. baumannii* Multi-Drug Resistant (MDR) or Extensively Drug-Resistant (XDR) (Vrancianu et al., 2020), therefore, our results are according to its resistance rate profile and are encouraging, as it belongs to the Critical priority group. Besides, no differences were found for *N. gonorrhoeae*, which has developed a wide variety of resistance mechanisms that make treatment difficult, and the same is true for *Shigella* spp., for instance, beta-lactamases, efflux pumps, and mutations in genes related to key proteins for antibiotic binding, among others., The not-listed bacteria, acting like external control, showed no considerable changes, which makes sense due to their scarcely resistant behaviour. *Clostridium difficile* and *Rickettsia* spp. statuses are consistent with their antibiotic sensitivity profile. *Clostridium difficile* has the ability to sporulate and form biofilms, allowing it to evade elimination and cause recurrences, although this is not a classic genetic resistance mechanism. *Rickettsia* spp. lack of resistance, which is attributed to its obligate intracellular cycle and absence of significant horizontal gene transfer, and remains fully susceptible. Finally, *C. trachomatis* presented a small significant difference, but it has not shown much stable clinical resistance to antibiotics. It can also form an intracellular “persistence” state where it tolerates antibiotics without genetic changes.

When the New Treatments were analyzed by year, *A. baumannii* rates showed a decrease followed by an increase, and also an increase in the reports on new treatments for *Shigella* spp. and *N. gonorrhoeae* was observed at the end of the 10 years (Figure 10). This is encouraging for the three bacteria mentioned according to their category in the alert (*A. baumannii*, Critical; *N. gonorrhoeae,* High; *Shigella* spp., Medium), and it can be interpreted as an intent to respond to the WHO alert.

On the other hand, the unlisted bacteria did not show significant changes, which could mean that in recent years, research into potential therapies has focused on the bacteria mentioned by the WHO, although high rates of reports were detected for *C. difficile*. The analysis by period supports these findings, since *A. baumannii*, *N. gonorrhoeae, Shigella* spp. and also *C. difficile* exhibited significant differences, with increased rates in the post-alert periods (Figure 11). This is important to *A. baumannii* since its resistance has increased, particularly to beta-lactams, aminoglycosides, fluoroquinolones, and most recently to carbapenems. For *N. gonorrhea,* there are few available antimicrobial treatment options, based on ceftriaxone and azithromycin, and *Shigella* strains have high resistance levels to ampicillin, trimethoprim-sulfamethoxazole, and fluoroquinolones; hence, these positive results show a promising breakthrough in the fight against antibiotic resistance (Bennett et al., 2020). Also, for *C. difficile*, due to the clinical relevance of this microorganism. It developed resistance to fluoroquinolones due to a point mutation that characterized a hypervirulent strain that caused severe outbreaks in the 2000s. On the contrary, investigations on new therapies for *C. trachomatis* and *Rickettsia* spp. did not suffer any change, and this is a cause for concern, especially for the first species, given its high re-infection rate (Xu et al., 2023) and the health problems it represents due to mother-to-child transmission, which leads to the search for safer treatments during pregnancy. As it was described previously, *Rickettsia* spp. have not shown significant resistance to doxycycline (the treatment of choice) and remain fully susceptible. However, sulfonamides can worsen the infection by increasing rickettsial proliferation (a paradoxical mechanism). In this area, it is important to point out that the development of new treatments is usually more time-consuming than the finding of new resistant strains, and the whole effect of the alert on the new treatments could be better appreciated in a 10-year window.

On the type of treatments, the highest percentage was New combinations of antibiotics (Figure 12), which is not surprising, given that one of the most widely used strategies in the clinical area is to administer more than one antibiotic to enhance or synergize the effects against resistant microorganisms. Physicochemical treatments appear in second place, and after a review, we hypothesize that LLMzCor, included in this category also combines therapies, which may belong to more than one subcategory, i.e., nanoparticles of natural products activated by light. In this sense, an improved performance can be obtained after reviewing subcategory classifications by redefining the prompt. Antivirulence strategies (third place) emerge as a promising alternative to reduce the virulence instead of killing the pathogen, which is desirable to decrease mortality rates. Peptides are prevailing over Designer drugs and Bacteriophages; however, the latter therapeutic alternative presents encouraging results. Even though it does not stand out among the most reported therapies, the historical importance of Natural Products, specifically phytochemicals, as prototypes for Designer drugs must be highlighted, as well as the potential of botanicals -a complex matrix composed of several molecules-for treatment of microorganism infections, since their components can synergize and exert enhanced effects. All these results showcase a diverse approach to combating antibiotic resistance.

Prevention can be a goal in the fight to stop the spread of infectious diseases. Therefore, the last point of our analysis was the state of advance in Immunization. In all the bacteria analyzed, the number of New immunization strategies or proposals is low, in comparison with the total number of papers published, and they remained below 10%. The highest rates over the 10 years were determined for *Shigella* spp., *C. difficile* and *C. trachomatis*, and this slightly greater interest in developing immunizations could be explained by their transmission routes and the impact they have on health The low percentages of remaining bacteria invite us to reflect on the promotion and exploration of this area of research (Figure 13). When differences from pre-to post-alert period were examined, only *N. gonorrhoeae* and *A. baumannii* exhibited positive changes. Fortunately, it means a small growing trend for both in the post-alert period. The percentages of *Shigella* spp., *C. difficile* and *C. trachomatis*, although they were the most prominent, remained unchanged (Figure 14). Nevertheless, even when two WHO listed bacteria presented statistically significant differences, there is no clear and strong tendency over the years nor between periods pre- and post-alert, with immunization being the weakest area in the fight against the untreatable bacteria. Probable causes of this lack of new candidates are the high multiplicity of serotypes, the genetic variability, the different strategies of evading the immune system, and the weak or short-lived immune response generated. As with treatments, it takes between 10 and 20 years to develop new vaccines. Besides, there is a lack of adequate methods to measure cost-effectiveness in potential vaccines, since their impact on resistance is not usually evaluated, and their estimated value in this matter is lower (Micoli et al., 2021).

## 5 Conclusion, limitations and future directions

Our work provides a perspective on the advances published on certain bacteria, some included in the alert issued by the WHO, and others not included but clinically relevant, while also providing indicators on the impact of this alert on research for the scientific community. While at first glance, the total number of publications for all bacteria increased after the 2017 alert, primarily for the listed bacteria, a selective analysis of reports of resistant strains reveals that, fortunately, there was no increase in the published rate for these strains since they have been reported regularly. However, when the relative data on innovative therapies in any phase of development are analyzed, significant but moderate increases were observed. The most notable is the new combination of already used antibiotics, which is a reasonable strategy given their known characteristics (knowledge of their safety profile and mechanism of action, already approved, commercial availability, among others) and the time required to develop new therapies. Even when these findings offer hope, it would be desirable to increase research into other types of potential new treatments so that more become approved therapies in the future. This can be extended to new immunization approaches, since the results obtained presented a still underdeveloped scenario, probably caused by the inherent characteristics of each microorganism, the long-time development for their approval and commercialization (which could be accelerated as happened with SARS-CoV-2 vaccines) and the low importance given to them in the face of reducing the incidence of infections with the consequent reduction in the use of antibiotics, and consequently, of AR, among others factors. Besides the education of the society and the alert of the danger represented by the listed bacteria, immunization plays an important role.

On the other hand, we have developed LLMzCor, an AI-based tool to analyse and follow the advances in scientific publications. Complementarily, the AI assistant proved to be a valuable tool for enhancing the efficiency of SRs, and allows automation of analysis of a considerable amount of data for decision-making. Its implementation can shift the focus of bibliographic reviews efficiently and extract valuable insights that can guide future research directions. Nevertheless, some limitations of this study outside the model are the inclusion of articles only in English and those reported in PubMed. Regarding the model, one of the main limitations that remain in the use of language models such as LLMzCor is the generation of inaccurate or unverified content, commonly referred to as hallucinations by the AI. These errors can occur even when the model is trained on large volumes of data, as its ability to generate coherent text is not always matched by factual accuracy. This risk increases when working with scientific literature that is incomplete, ambiguous, or poorly structured. Therefore, expert human curation becomes essential—not only to validate automated classifications but also to detect when the model’s output lacks scientific rigor or is based on low-quality data. Additionally, relying solely on abstracts as the source of information limits the depth of analysis, since many experimental details, results, and methodological limitations are only reported in the full text. Future versions of the model could be integrated with databases that provide automated access to full-text documents, with systems capable of detecting when an abstract lacks sufficient evidence for reliable classification, or integrating more robust preprocessing methods, domain-specific fine-tuning, and hybrid pipelines combining AI predictions with human validation. Establishing minimum quality standards for scientific abstracts could also enhance the reliability of LLM-assisted tools like LLMzCor. In order to include and organize the increasing literature for further analyses, the development of innovative tools such as machine learning would be necessary. These powerful tools could accelerate bibliographic research and give scientists a broader view of published results. They could also be used to improve and accelerate the application of the right treatment for each bacterial infection.

In light of the limited progress in developing new treatments or immunization strategies for the listed bacteria, we strongly advocate a more assertive WHO alert accompanied by comprehensive medium- and long-term action plans with robust support from national governments and international institutions. These plans should include increased funding for scientific research, incentives for international collaboration, stronger awareness campaigns, preparation of all personnel in the health system, and facilitation of interaction between academia and the private sector.

In order to include and organize the increasing literature for further analyses, the development of innovative tools such as ML would be necessary. These powerful tools could accelerate bibliographic research and give scientists a broader view of published results. They could also be used to improve and accelerate the application of the right treatment for each bacterial infection.

## Data Availability

The raw data supporting the conclusions of this article will be made available by the authors, without undue reservation.
