# Supplementary material for: The WHO’s critical bacteria list: scientific response eight years after its implementation and development of an AI-based tool for its monitoring
Source: Front Pharmacol. 2025 Sep 11;16:1633382. doi: 10.3389/fphar.2025.1633382 (PMC12461225; doi:10.3389/fphar.2025.1633382)
Supplement: Supplementary file 1 [file DataSheet1.docx]

**Supplementary Material**

**Eight years later: scientific response to the WHO's critical bacteria list and development of LLMzCor for their monitoring**

Juan Eduardo Robledo Almonacid^1,2^, Christian Lombardo^3^, Mariana Romano^4^, Agustina Quiroga^4^, Paula Cambuli Bianchi^5^, Mauricio Hualpa^6^, Constanza Giai^4^, Xiomara María Oviedo^7^, Ramiro Alejo Salgado Mansur^1,8^, Mariana Guadalupe Vallejo^1,8†*^, Cristián Andrés Quintero^4,5†*^

^1^ Farmacognosia, Departamento de Ciencias Farmacéuticas, Facultad de Ciencia Químicas, Universidad Nacional de Córdoba, Córdoba, Argentina

^2^ Instituto de Investigaciones en Ciencias de la Salud-Consejo Nacional de Investigaciones Científicas y Técnicas, Córdoba, Argentina

^3^ Facultad de Ciencias Exactas y Naturales, Universidad de Buenos Aires, Buenos Aires, Argentina

^4^ Laboratorio de Biología Molecular y Celular-BioCyM. Universidad Juan Agustín Maza, Mendoza, Argentina.

^5^ INBIOMED-UM Facultad de Ciencias Médicas, Universidad de Mendoza, Mendoza, Argentina.

^6^ Obra Social de Empleados Públicos-OSEP-Mendoza, Argentina

^7^ Facultad de Ciencias Exactas, Físicas y Naturales. Universidad Nacional de La Rioja, La Rioja, Argentina

^8^ Unidad de Investigación y Desarrollo en Tecnología Farmacéutica-Consejo Nacional de Investigaciones Científicas y Técnicas, Córdoba, Argentina

†These authors share last authorship

* Correspondence:
Mariana Guadalupe Vallejo
mariana.vallejo@unc.edu.ar

Cristián Andrés Quintero

cristian.quintero@um.edu.ar

**Formula 1.** Difference between pre-alert publications and post-alert publications, expressed as percentage of pre-alert publications.

[(No. of publications of bacterium “X” in the post-alert period - No. of publications of bacterium “X” in the pre-alert period)/No. of publications of bacterium “X” in the pre-alert period] x 100

**Formula 2.** Rate of publication in pre- and post-alert period regarding each bacterial species.

(No. of publications of bacterium “X” in the pre-alert period/No. of total publications of all bacteria in the pre-alert period) x 100

*Idem for post-alert period.*

**Formula 3.** Rate of publication of resistant strains per year regarding each bacterium.

(No. of publications of resistant strains of bacterium “X” in a year/No. of total publications of bacterium “X” in a year) x 100

**Formula 4.** Rate of publication of resistant strains in pre- and post-alert period regarding each bacterium.

(No. of publications of resistant strains of bacterium “X” in the pre-alert period/No. of total publications of bacterium “X” in the pre-alert period) x 100

*Idem for post-alert period.*

**Formula 5.** Rate of publication of new treatments per year regarding each bacterium.

(No. of publications of new treatments for bacterium “X” in a year/No. of total publications of bacterium “X” in a year) x 100

**Formula 6.** Rate of publication of new treatments in pre- and post-alert period regarding each bacterium.

(No. of publications of new treatments for bacterium “X” in the pre-alert period/No. of total publications of bacterium “X” in the pre-alert period) x 100

*Idem for post-alert period.*

**Formula 7.** Rate of publication of new immunization strategies per year regarding each bacterium.

(No. of publications of new immunization strategies for bacterium “X” in a year/No. of total publications of bacterium “X” in a year) x 100

**Formula 8.** Rate of publication of new immunization strategies in pre- and post-alert period regarding each bacterium.

(No. of publications of new immunization strategies for bacterium “X” in the pre-alert period/No. of total publications of bacterium “X” in the pre-alert period) x 100

*Idem for post-alert period.*

**TABLE 1** Proportion of publications of each bacterium with respect to the total number of publications in each period

| Species | % publications pre | % publications post | % difference | CI | Z | p |
| --- | --- | --- | --- | --- | --- | --- |
| *Acinetobacter*  *baumannii* | 19.11% | 23.35% | 4.24% | -0.051, -0.034 | -9.946 | 0.000 |
| *Neisseria*  *gonorrhoeae* | 9.01% | 9.73% | 0.72% | -0.013, -0.001 | -2.373 | 0.018 |
| *Shigella* spp. | 11.71% | 12.79% | 1.08% | -0.017, -0.004 | -3.157 | 0.002 |
| *Rickettsia* spp. | 17.98% | 17.88% | -0.10% | -0.007, 0.009 | 0.254 | 0.800 |
| *Chlamydia*  *trachomatis* | 13.07% | 11.54% | -1.53% | 0.009, 0.022 | 4.491 | 0.000 |
| *Clostridium*  *difficile* | 29.12% | 24.71% | -4.41% | 0.035, 0.053 | 9.609 | 0.000 |
